# The Transcription Factor *VpxlnR* Is Required for the Growth, Development, and Virulence of the Fungal Pathogen *Valsa pyri*

**DOI:** 10.3389/fmicb.2022.784686

**Published:** 2022-03-03

**Authors:** Feng He, Alex-Machio Kange, Jie Yang, Jiaxin Xiao, Rongbo Wang, Lu Yang, Yifan Jia, Zheng Qing Fu, Yancun Zhao, Fengquan Liu

**Affiliations:** ^1^Jiangsu Key Laboratory for Food Quality and Safety-State Key Laboratory Cultivation Base of Ministry of Science and Technology, Institute of Plant Protection, Jiangsu Academy of Agricultural Sciences, Nanjing, China; ^2^College of Life Sciences, Anhui Normal University, Wuhu, China; ^3^Department of Agriculture and Natural Resource, Bomet University College, Bomet, Kenya; ^4^Fujian Key Laboratory for Monitoring and Integrated Management of Crop Pests, Fuzhou, China; ^5^Department of Biological Sciences, University of South Carolina, Columbia, SC, United States

**Keywords:** fungi, transcription factor, pear pathogens, virulence, stress

## Abstract

Pears (*Pyrus* sp.) are widely cultivated in China, and their yield accounts for more than 60% of global pear production. The fungal pathogen *Valsa pyri* is a major causal agent of pear canker disease, which results in enormous losses of pear production in northern China. In this study, we characterized a Zn_2_Cys_6_ transcription factor that contains one GAL4 domain and a fungal-trans domain, which are present in VpxlnR. The *vpxlnR* gene expression was upregulated in the invasion stage of *V. pyri*. To investigate its functions, we constructed gene deletion mutants and complementary strains. We observed that the growth of the *vpxlnR* mutants was reduced on potato dextrose agar (PDA), Czapek plus glucose or sucrose compared with that of the wild-type strain. Additionally, *vpxlnR* mutants exhibited loss of function in fruiting body formation. Moreover, *vpxlnR* mutants were more susceptible to hydrogen peroxide (H_2_O_2_) and salicylic acid (SA) and were reduced in their virulence at the early infection stage. According to a previous study, VpxlnR-interacting motifs containing NRHKGNCCGM were searched in the *V. pyri* genome, and we obtained 354 target genes, of which 148 genes had Clusters of Orthologous Groups (COG) terms. PHI-BLAST was used to identify virulence-related genes, and we found 28 hits. Furthermore, eight genes from the 28 PHI-BLAST hits were further assessed by yeast one-hybrid (Y1H) assays, and five target genes, salicylate hydroxylase (VP1G_09520), serine/threonine-protein kinase (VP1G_03128), alpha-xylosidase (VP1G_06369), G-protein beta subunit (VP1G_02856), and acid phosphatase (VP1G_03782), could interact with VpxlnR *in vivo*. Their transcript levels were reduced in one or two *vpxlnR* mutants. Taken together, these findings imply that VpxlnR is a key regulator of growth, development, stress, and virulence through controlling genes involved in signaling pathways and extracellular enzyme activities in *V. pyri*. The motifs interacting with VpxlnR also provide new insights into the molecular mechanism of xlnR proteins.

## Introduction

Pear is the third most highly produced fruit in China. Valsa canker disease is one of the most destructive diseases in most orchards of northern China. The disease is caused by the fungal pathogen *Valsa pyri*, which belongs to Ascomycetes in the Valsaceae family (Sordariomycetes, Diaporthales; Yin et al., 2015). This fungus can infect pear trees from natural wound sites on the bark and then form cankers, which result in great yield loss or tree death [3, 4]. Although *Valsa mali* and *V. pyri* are similar species, they diverged 5 million years ago (Wang et al., 2014). *Valsa pyri* is a necrotrophic pathogen that can penetrate the phloem and xylem (Yin et al., 2015). Previously, it was shown that transcription factors (TFs), cell wall-degrading enzymes, and genes involved in nitrogen metabolism might be important for the virulence and growth of *V. pyri* strains (He et al., 2018; Xu et al., 2018). TFs, especially fungal-specific TFs, function as important regulators in fungal pathogens. However, very few studies have been conducted to investigate the roles of TFs in the pathogenesis of *V. pyri*.

Transcription factors can control the transcript levels of many target genes (Cho et al., 2013; He et al., 2016; Luo et al., 2016; Ishikawa et al., 2018; Oka et al., 2019). However, each gene can also be regulated by different TFs (Aro et al., 2001; Ishikawa et al., 2018). TFs and target genes constitute a network to regulate epigenetic modification, cell growth, cell differentiation, and stress responses (de Vries et al., 1999; Rauscher et al., 2006; Choi et al., 2009; Cho et al., 2013; Wu et al., 2018; Feng et al., 2020). To control gene expression, TFs generally possess one or more typical DNA-binding domains, and they are activated by themselves or other enzymes to bind promoter regions and induce mRNA transcription (Shelest, 2017; Ishikawa et al., 2018). Recently, as the full genomic sequences of more fungi became available, fungal TFs have been well characterized in different studies. According to a recent report, there are approximately 80 TF families in fungi, and many of them are fungus-specific TFs (Shelest, 2017). Fungus-specific TFs generally contain a typical fungal-trans domain, most of which contain other Zn_2_Cys_6_ clusters, and only a small portion of fungus-specific TFs contain C2H2 Zn fingers (MacPherson et al., 2006; Shelest, 2017). There are more than 100 fungus-specific TFs in most filamentous fungi (Shelest, 2017; He et al., 2018). Interestingly, many Zn_2_Cys_6_ TF orthologues exhibit various expression levels in different fungi, suggesting that they may have different roles in these fungi (He et al., 2018). Moreover, many TFs have been identified in many pathogenic fungi, and their roles and regulated genes have been well studied (Guo et al., 2011; Katz et al., 2013; Lu et al., 2014; He et al., 2016; Wu et al., 2018). For example, homeobox TFs are essential for conidiation and appressorium development (Kim et al., 2009). Hsf1 is a critical regulator of virulence traits (Veri et al., 2018), and VmSeb1 regulates development in *V. mali* (Wu et al., 2018), while VdMcm1 controls conidiation, microsclerotium formation, pathogenicity, and secondary metabolism (Xiong et al., 2016).

Although there are a large number of fungus-specific TFs, only a few are involved in virulence (Abe et al., 2007; Choi et al., 2009; Zhao et al., 2011; Cho et al., 2013; He et al., 2016; Wu et al., 2018). In previous studies, the fungus-specific TF AbPf2 and its orthologues were found to be involved in regulating fungal development, metabolism, and virulence in *Alternaria brassicicola*, *Verticillium dahlia*, and *Parastagonospora nodorum* (Cho et al., 2013; Luo et al., 2016; Rybak et al., 2017; Zhang et al., 2018). Due to the similar consensus sequences in the orthologues in various fungi, they generally have similar functions. Nevertheless, they also exhibit some unique roles in different isolates. For example, EBR1 orthologous gene FOXG_05408-knockout mutants in *Fusarium oxysporum* f. sp. lycopersici showed reduced virulence compared with the ebr1-deletion mutant in the PH-1 strain. These results indirectly proved the hypothesis that abundant Zn_2_Cys_6_ TFs may function in different processes and exhibit diverse functions in different fungal strains (Zhao et al., 2011; He et al., 2018). To control various aspects of fungal lifestyle, Zn_2_Cys_6_ TFs were previously reported to bind DNA motifs containing a CGG triplet (Cho et al., 2013; Luo et al., 2016; Raulo et al., 2016; Ishikawa et al., 2018). Thus, with high-throughput sequencing, such as transcriptome analysis or ChIP-seq, different motifs were found to interact with Zn_2_Cys_6_ TFs. In brief, Zn_2_Cys_6_ TFs bind a great number of genes to control fungal life.

XlnR, which contains one Zn_2_Cys_6_ cluster, has been characterized in several filamentous fungi and has vital roles in sugar metabolism in fungi (van Peij et al., 1998; MacPherson et al., 2006; Rauscher et al., 2006; Fujii et al., 2014). Its orthologues exhibit similar roles in several fungi. Thus, they can also regulate different genes in several fungi (Hasper et al., 2000; Calero-Nieto et al., 2007; Klaubauf et al., 2014; Llanos et al., 2019). The common function of XlnR in *Aspergillus* spp., *Trichoderma reesei*, *Fusarium species*, *Magnaporthe oryzae* (*Pyricularia oryzae*), and *Neurospora crassa* is to control xylanolytic and cellulolytic gene expression (Marui et al., 2002; Rauscher et al., 2006; Tamayo et al., 2008; Sun et al., 2012; Battaglia et al., 2013). XlnR regulates gene expression by binding to the CGGNTAAW motif as a monomer and by binding to the TTAGSCTAA motif as a dimer in *A. oryzae* (Ishikawa et al., 2018). These studies indicate that XlnR could function as a monomer and a dimer to control gene expression. Until now, the xlnR TF was only found to be involved in early infection by *F. graminearum*. However, its orthologous genes in *Valsa* species have not been identified. In this study, we discovered its roles in the pathogenicity of *V. pyri* and explored its molecular mechanism in controlling gene expression.

## Materials and Methods

### VpxlnR Identification and Its Expression Pattern in the *Valsa pyri* Infection Stage

The TF open reading frame (ORF) sequence was obtained from the RNA-seq database (He et al., 2018), and the protein sequences were predicted using ORF finder.^1^ The hypothetical protein was characterized using BlastP, and its orthologues, including VmxlnR (KUI73112.1), hypothetical proteins (ROV95313.1, ROW10257.1, KAB5560083.1, XP_030979480.1, XP_016619423.1, and XP_001394612.2), TlxlnR (KAF3060643.1), BbxlnR (KAF1730510.1), MpxlnR (KLU82989.1), VlxlnR (KAG7119801.1), VdxlnR (XP_009650488.1), FoxlnR (RKL21210.1), and CgxlnR (KAF3801118.1), were acquired from the NCBI website. Similar to VpFSTF1, a phylogenetic tree was constructed using the hypothetical protein and its orthologous sequence by MEGA 7.0 (Kumar et al., 2016) and the hypothetical protein named VpxlnR. Furthermore, all protein sequences were submitted to the pfam database^2^ by researching the protein domain, and later, the domains were drawn according to the research results. The transcript levels were evaluated as described previously (Kange et al., 2020).

### Generation of Deletion Mutants for VpxlnR and Complementary Strains

DNA sequences approximately 2 kb upstream or downstream were acquired through BLASTn to *V. pyri* genomes, and primers 20 bp from a cassette containing the hygromycin phosphotransferase (*hph*) gene were designed. The primer pair of the cassette was also set with an 18–20 bp joint of upstream or downstream DNA sequences. We extracted Vp297 genomic DNA through the cetyltrimethylammonium bromide (CTAB) protocol (Umesha et al., 2016). Then, the upstream sequence, *hph* cassette and downstream sequence were amplified using the 1/2, 3/4, and 5/6 primer pairs (Supplementary Table S5), respectively. Then, the PCR products were purified using a PCR kit. Based on a previous study (He et al., 2016), the VpxlnR allele construct was amplified using the upstream sequence, *hph* cassette, and downstream sequence at a ratio of 1:3:1, and a 1/6 primer pair and primers were designed (Supplementary Figure S2; Supplementary Table S5). The VpxlnR allele was purified, and the purified product was transferred to the protoplast of the wild-type strain Vp297 using an improved polyethylene glycol (PEG)-mediated fungal transformation protocol (He et al., 2016). The VpxlnR deletion mutants were obtained on PDA medium after adding 50 mg/l hygromycin B, and approximately 120 primary transformants were acquired. To confirm whether the gene was deleted in these transformants, a partial DNA fragment from the ORF of VpxlnR was amplified using primer 7/8 primer pairs, and further, the *hph* fragment was amplified using 9/10 primer pairs (Supplementary Figure S2; Supplementary Table S5). Additionally, allele site replacement was ascertained using the 9/12 and 11/10 primer pairs (Supplementary Figure S2; Supplementary Table S5). In addition to genomic PCR, quantitative reverse transcription PCR (RT–qPCR) was carried out to evaluate the expression level of VpxlnR in the candidate mutants using the 15/16 primer pairs (Supplementary Figure S2; Supplementary Table S5).

We selected the vector pFL2 (Li et al., 2015a) as the overexpression plasmid, which was driven by the strong promoter RP27. The VpxlnR gene was amplified using primer pair 17/18, which contains a 20 bp sequence joint from the pFL2 plasmid, and then purified using a PCR kit (AxyPrep PCR Cleanup Kit, Suzhou, China). The resulting PCR product was constructed into XhoI-digested pFL2 through an one-step clone kit (Vazyme, Nanjing, China), and the recombinant vector was then transformed into *Escherichia coli* (DH5a cells). Later, the vector was extracted using a method described by a previous study (Kange et al., 2020). The plasmids were assessed by sequencing (GenScript, Nanjing, China), and the correct plasmids were used for further study. Then, the plasmid was transferred to protoplasts of the mutant m-56 by PEG-mediated transformation (He et al., 2016). The transformants were screened on PDA amended with 75 mg/L G418, and several positive transformants were further confirmed by genomic PCR and RT–qPCR.

### Mycelial Growth and Fruiting Body Formation

Mycelial growth of the deletion mutants was characterized on PDA or Czapek media with different carbon sources, including glucose, sucrose, cellulose, sodium, pectin, and the control consisting of null sugar. Fruiting bodies were induced in the wild-type and complementary strains as reported in a previous study (He et al., 2016). The colony sizes of the wild-type Vp297, mutant and complementary strains grown on PDA media were measured at 36 h, and images were captured at the same time. Additionally, the colony sizes of the strains on Czapek media amended with glucose, sucrose, pectin, cellulose sodium, and null sugar were measured at 48 h, and later, these data were calculated using GraphPad prism 7.0. Every treatment was replicated on at least three agar plates.

### Pathogenicity Assay

Pathogenicity assays were performed according to a previous study (Kange et al., 2019). Lesion development on inoculated leaves was observed daily, and images were captured at 3 and 5 days post-inoculation (dpi). Lesion lengths on inoculated branches were also observed daily, and images were captured at 2 and 4 dpi. Each experiment was duplicated with eight leaves and 13 branches, and the lesion size was calculated using GraphPad prism 7.0.

### Host Mimic Stresses

Mycelial growth on PDA under H_2_O_2_ and SA stresses was used to assess the sensitivity of the mutants to host mimic stresses. The protocol used was similar to that used in a previous study (Kange et al., 2020). Colony sizes were measured at 36 h, and images were captured at the same time. Each experiment was repeated on at least three plates.

### VpxlnR-Binding Promoter and Virulence-Related Gene Prediction

We first obtained all of the genes predicted in a previous study (Yin et al., 2015). Then, 2 kb predicted promoter sequences upstream of the initial gene codes were acquired. Later, according to the CGG triplet-containing DNA motif predicted by previous studies, we searched all predicted promoter sequences using Regular Expression, and candidate promoters were further obtained (Cho et al., 2013). Moreover, candidate genes controlled by these promoters were acquired, and their DNA binding sites were also predicted using the Berkeley Drosophila Genome Project (BDGP, https://www.fruitfly.org/seq_tools/promoter.html). Additionally, their expression levels in the *V. pyri* infection stage were predicted according to *V. pyri* transcriptome analysis (He et al., 2018). The genes that were upregulated in the infection stage were selected, and later, their COG functional annotation was analyzed according to the gene annotation in *V. pyri* transcriptome analysis (He et al., 2018). Based on the gene annotation, we drew a COG term enrichment graph. Furthermore, to explore genes participating in virulence, all of the candidate genes were blasted to the PHI database, and genes with PHI hits were used for further identification.

### Yeast One-Hybrid Assay

Based on COG annotation and PHI analysis, we selected eight genes, including salicylate hydroxylase (VP1G_09520), serine/threonine-protein kinase (VP1G_03128), alpha-xylosidase (VP1G_06369), G-protein beta subunit (VP1G_02856), acid phosphatase (VP1G_03782), serine/threonine-protein kinase KIN28 (VP1G_04075), serine/threonine-protein kinase GCN2 (VP1G_10966), and putative phosphotransferases (VP1G_03516), for the yeast one-hybrid (Y1H) assay. Of these genes, a 1,500–2,000 bp DNA sequence upstream of the start codon of each gene was amplified using genomic PCR, and then, the PCR products were purified using a PCR kit. The VpxlnR gene was ligated to a linearized pGADT7 vector. The promoter region of each gene was cloned into the pHIS2 vector, which was linearized by SmaI digestion. Positive clones were further confirmed by sequencing, and plasmids were obtained from the GenScript Company (Nanjing, China). The pAD::VpxlnR vector and the pHIS2::promoter vector were cotransformed into AH109 Gold yeast competent cells. Positive clone screening and confirmation were performed in accordance with a previous study (Kange et al., 2019).

### Expression Levels of the Target Genes Controlled by VpxlnR

According to the Y1H results, we analyzed the transcript levels of eight genes (VP1G_06369, VP1G_03128, VP1G_03782, VP1G_09520, and VP1G_02856) that were directly controlled by VpxlnR and three genes (VP1G_04075, VP1G_03516, and VP1G_10966) with negative results of Y1H. The RT–qPCR protocol and analysis of the results were the same as those in a previous study (Kange et al., 2019). Each experiment was repeated at least three times.

## Results

### Phylogenetic Analysis and Expression Pattern of VpxlnR in *Valsa pyri*

**Figure 1:**
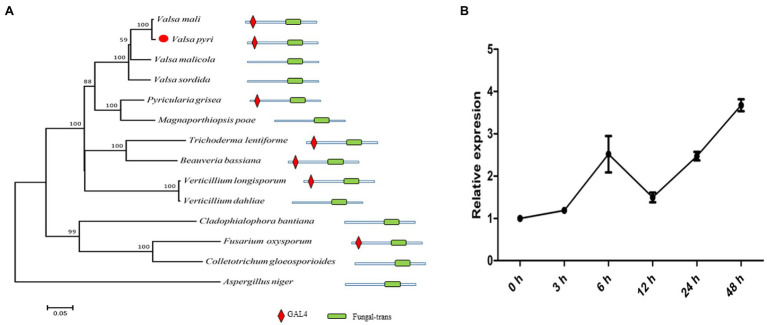
VpxlnR characterization and its expression pattern during infection. **(A)** Phylogenetic tree of xlnR orthologues in different fungi. The VpxlnR gene sequence was obtained from *Valsa pyri* transcriptomics data, and sequences of xlnR orthologues were downloaded from the NCBI database. Sequence alignment was carried out using ClustalW, and a phylogenetic tree was generated *via* the neighbor-joining method using MEGA 7.0 software. The confidence values above the nodes were acquired from 1,000 bootstrap analyses. The domain architecture of VpxlnR and its orthologues were searched with the Pfam database. The domains were manually drawn according to the search results. **(B)** Transcript levels of *VpxlnR* during *V. pyri* infection. The relative expression levels of *Vpxl*nR in *V. pyri* were quantified by quantitative reverse transcription PCR (RT–qPCR) and normalized to the transcript levels of the *V. pyri* actin gene. Fold changes were calculated by the 2^-∆∆Ct^ approach. Each experiment was performed three times.

In previous studies, we found that many genes in *V. pyri* were annotated as xlnR homologues through RNA-Seq analysis (He et al., 2018), and its unigene (c14467) was reidentified through *de novo* assembly using transcriptome data. The gene ORF with a length of 2,625 bp was predicted. Alignment using the unigene and genomic gene sequences was performed. As a result, we found that the gene contains four introns; however, the genome assembly was missing a 12-bp sequence, which might cause the prediction using genomics and transcriptomics to vary (Supplementary Figure S1). The xlnR-like protein predicted using transcriptomics contains a Zn_2_Cys_6_ zinc cluster and fungal-trans domain and is highly similar to other xlnRs in other fungal species, especially *Valsa mali* (Figure 1A), so we named it VpxlnR. Moreover, the domains of VpxlnR orthologues were analyzed. In most selected fungi, orthologues have similar domains consisting of a Zn_2_Cys_6_ cluster and a fungal-trans domain, but several VpxlnR orthologues lost the Zn_2_Cys_6_ cluster in *Valsa malicola*, *Valsa sordida*, *V. dahlia*, *Colletotrichum gloeosporioides*, and *Aspergillus niger* (Figure 1A), which may be caused by alternative splices or natural selection. These results suggested that VpxlnR is a fungus-specific transcription factor.

To further study the transcript levels of *VpxlnR*, total RNA samples of mycelia and mycelial infection pear bark were acquired [0–48 h post-inoculation (hpi)], and RT–qPCR was performed. At the early infection stage, *VpxlnR* expression was upregulated until 6 h after inoculation, but its transcript levels were downregulated at 12 h after inoculation. Furthermore, its transcript level restored the upregulation at the late infection stage (Figure 1B). These results suggest that the expression of the *VpxlnR* gene can be induced by pear plants.

### Generation of *VpxlnR* Deletion Mutants and Complementary Strains

To determine the functions of VpxlnR in *V. pyri*, two *VpxlnR* deletion mutants were generated using PEG-mediated transformation (Supplementary Figure S2). The deletion mutants were selected on PDA medium containing 50 mg/L hygromycin B. The *VpxlnR* gene in the two mutants was successfully replaced by a hygromycin-resistant cassette, which we named m-7 and m-56 (Supplementary Figure S2A). Furthermore, we constructed two complementary strains using protoplasts of the m-56 mutant as the target cells by PEG-mediated transformation, and the PCR amplification results showed that the *VpxlnR* gene was successfully amplified in these complementary strains (Supplementary Figure S2A). To examine the expression levels of *vpxlnR* in these mutants and complementary strains, RT–qPCR assays were performed, and the results showed that the expression of the *vpxlnR* gene was not detected in the two mutants, while the transcript levels of *vpxlnR* were normal in the two complementary strains (Supplementary Figure S2B). Taken together, these data indicate that we successfully acquired *vpxlnR*-knockout mutants and complementary stains of *V. pyri*.

### VpxlnR Controls Mycelial Growth and Fruiting Body Formation

**Figure 2:**
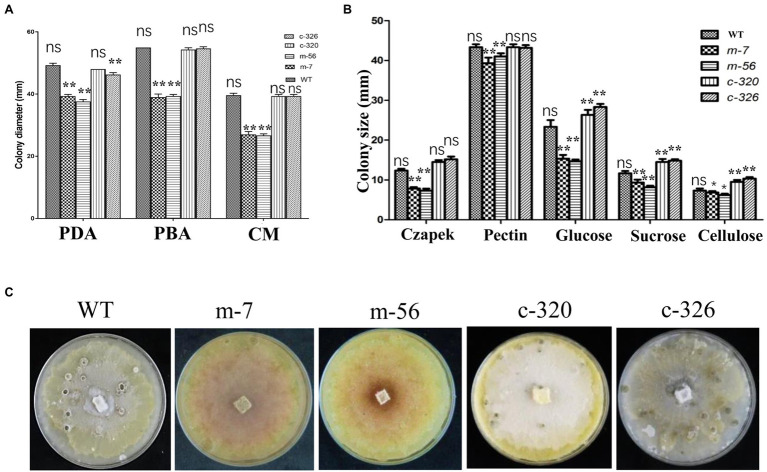
Characterization of growth and development of the *vpxlnR* mutants. **(A)** Colony growth on potato dextrose agar (PDA), pear branch agar (PBA), and CM media. Mycelial agar plugs of wild-type (WT), *vpxlnR mutants* (m-7 and m-56), and *VpxlnR* complementation (C-320 and C-326) were placed on PDA, PBA, and CM media. Colony diameter was measured at 36 h, and images were captured at the same time. **(B)** Radial growth of WT, *vpxlnR mutants*, and complementation on carbon sources. Colony diameters were measured at 36 h. Czapek’s medium supplemented with glucose, sucrose, pectin, and cellulose, and Czapek only served as the control. The plates were incubated at 25°C in the dark for 48 h. Typical images of the colonies were captured at 36 h. **(C)** Fruiting body formation. Fruiting bodies were induced under 16-h light/8-h dark for 20 days on PDA medium. Each strain was cultured on three agar plates. Images were captured at 20 days. In the image, ns indicates a value of *p* > 0.05, * indicates a value of *p* < 0.05, ** indicates a value of *p* < 0.01, and the statistical analysis was performed using two-way ANOVA.

To characterize the morphological features of the *vpxlnR* mutants, we recorded mycelial growth on PDA, pear branch agar (PBA), CM media, or Czapek media containing different carbon sources for fruiting body production. In the mutants cultured on both PDA, PBA, and CM media, 36 h later, the colony sizes of the *vpxlnR* mutants were significantly smaller than those of the wild-type (Vp297) and the *VpxlnR*-overexpressing complementary strains (Figure 2A). Because we added equal amounts of glucose to the three media, the growth ratios of the stains were similar on the three media. We speculate that the deletion of *VpxlnR* might weaken the ability of *V. pyri* to use carbon resources. To evaluate the abilities of *vpxlnR* mutants in the utilization of carbon resources, the mutants were grown on Czapek media with pectin, sucrose, glucose, and cellulose and with no sugar as the control. We found that the colony sizes of the mutants were obviously smaller than those of the wild-type or complementary strains (Figure 2B) on the five media. These results suggest that VpxlnR may determine glucose and sucrose assimilation, which results in a low growth ratio of the mutants. Furthermore, the mutants and other strains were cultured under light conditions. After 20 days, the mutants failed to produce fruiting bodies, while the wild-type and complementary strains produced fruiting bodies, indicating that VpxlnR affected fruiting body formation (Figure 2C). Taken together, these results demonstrated that VpxlnR plays important roles in the growth and development of *V. pyri*.

### VpxlnR Regulates Virulence of *Valsa pyri*

**Figure 3:**
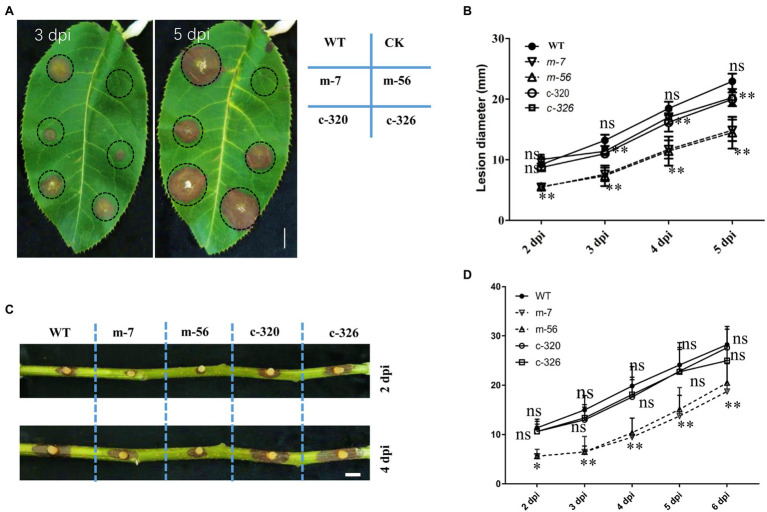
Effect of *VpxlnR* on virulence. **(A)** Virulence of the *vpxlnR* mutants on pear leaves. Mycelial agar plugs of the strains, including WT, *vpxlnR* mutants, and complementation, were inoculated on pear leaves. Photos were taken at 3 and 5 dpi, respectively. Lesion diameters were measured at 2–5 dpi. **(B)** Lesion diameters on pear leaves. Lesion diameters were collected on eight leaves. **(C)** Pathogenicity of the *vpxlnR* mutants on pear branches. Related strains were inoculated on wounded 1-year-old branches, and branches were incubated at 25°C in the dark. Branch images were captured at 2 and 4 dpi. Lesion diameters were measured at 2–6 dpi. **(D)** Lesion lengths on pear branches. Lesion lengths were obtained from 13 inoculated branches. Bar = 1 cm, ns indicates a value of *p* > 0.05, * indicates a value of *p* < 0.05, and ** indicates a value of *p* < 0.01; the statistical analysis was performed by two-way ANOVA.

To assess the effect of VpxlnR on virulence, mycelial agar plugs of the wild-type, mutant, and complementary strains were inoculated on detached pear leaves and branches. The diameters of the lesions on leaves were measured at 2, 3, 4, and 5 dpi. We found that the lesion sizes produced by the *vpxlnR* mutants were significantly smaller than those produced by the wild-type and complementary strains at 2–5 dpi (Figures 3A,B). Accordingly, the lesion length caused by the mutants on the inoculated branches was significantly smaller at each time point (2–6 dpi; Figures 3C,D). These results suggest that VpxlnR may play an important role in the determination of infection progression at the invasion stage on pear trees.

### The *vpxlnR* Mutants Are Sensitive to Host Mimic Stress in a Concentration-Dependent Manner

**Figure 4:**
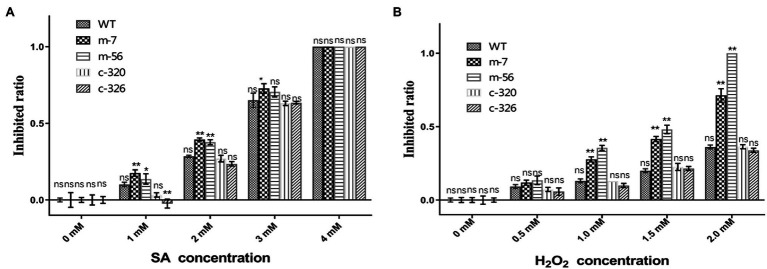
Susceptibility of the *VpxlnR* mutant to host mimic stresses. **(A)** Effect of SA stress on strain growth. Wild-type (WT), VpxlnR deletion mutant (m-7 and m-56), and complementation (C-320 and C-326) strains were grown on PDA medium supplemented with the indicated SA concentrations and incubated at 25°C for 36 h. **(B)** Impact of oxidative stress on colony growth. Strains were cultured on PDA medium supplemented with H_2_O_2_ and incubated at 25°C for 36 h. Each treatment was repeated three times, and colony diameters were recorded at 36 h. The data were recorded at 36 h. The statistical analysis was performed by two-way ANOVA, ns indicates a value of *p* > 0.05, * indicates a value of *p* < 0.05, and ** indicates a value of *p* < 0.01.

Pathogens must overcome stresses, especially reactive oxygen species (ROS) or immune signalling chemicals such as salicylic acid (SA), from plants to invade successfully. Because the pathogenicity of the *vpxlnR* mutants was significantly reduced on pear branches and leaves, they may be more sensitive to host immune responses such as ROS burst and SA accumulation. To evaluate whether the mutants could resist host-derived stresses, we recorded the growth status on PDA media amended with different concentrations of H_2_O_2_ or SA. With increasing SA concentration, the colony size of all of the strains was reduced at 36 h post-inoculation (hpi) on PDA. Compared with the wild-type strain, the mutants (m-7 and m-56) showed higher inhibited ratio on the plates adding 1.0 and 2.0 mM SA, and their growth was completely inhibited by 4.0 mM SA. However, the complementary strains restored deficiencies of the mutants and exhibited similar or lower inhibited ratio with increasing SA concentration (Figure 4A). These results indicate that VpxlnR has a large impact on SA stress. Similar to the SA stress assays, the mutants exhibited increased susceptibility to H_2_O_2_. With an increasing concentration of H_2_O_2_, the inhibited ratio of the mutants were greatly increased compared with those of the wild-type and complementary strains (Figure 4B). When the H_2_O_2_ concentration was 2 mM, mycelial growth of the m-56 mutant was completely inhibited. Similarly, the growth of the m-7 mutant was also significantly reduced, and its colony became abnormal, showing a thinner layer than the wild-type and the complementary strains (Figure 4B). These results indicate that VpxlnR is involved in H_2_O_2_ stress in *V. pyri*. Taken together, these results demonstrate that VpxlnR functions in response to overcoming host immunity to aid infection.

### Prediction of VpxlnR Binding Promoters

**Figure 5:**
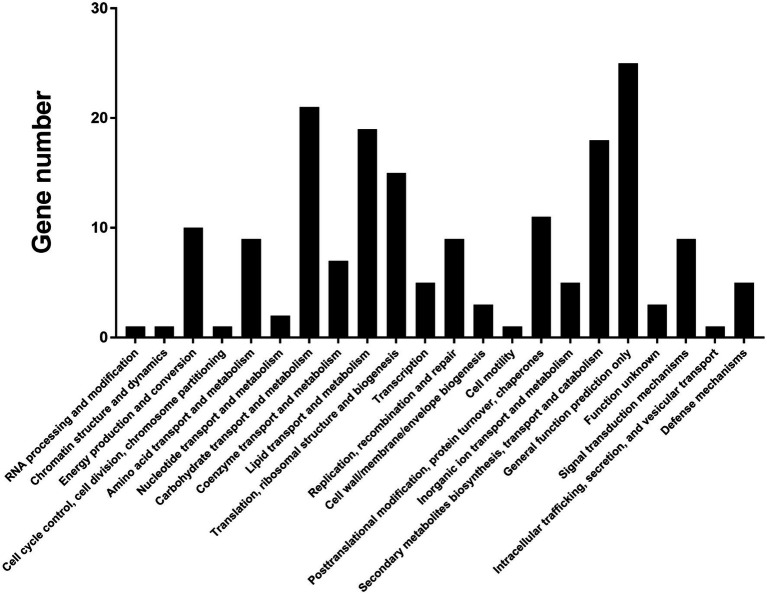
COG annotation of the predicted target genes. Approximately 148 target genes have COG terms. COG terms are indicated on the image. *X*-axis: COG classes. *Y*-axis: shown in the image.

To characterize genes controlled by VpxlnR, we obtained candidate promoter sequences (2000 bp upstream region from the initial codon of each gene) by searching CGG triplets or TTAGSCTAA in *V. pyri*. As a result, we found 354 promoters that have similar motifs, among which 268 promoters were present on the antisense strand, and 86 promoters were present on the sense strand (Supplementary Table S1). The genes downstream of each promoter were also obtained using Seqhunter 1.0, and the COG functional annotations were analyzed. A total of 148 genes had COG terms, and the most enriched terms were involved in “carbohydrate transport and metabolism,” “lipid transport and metabolism,” “secondary metabolite biosynthesis, transport, and catabolism,” and “general function prediction only” (Figure 5; Supplementary Table S2), which suggests that VpxlnR controls carbon resource utilization. However, there is limited information on the genes involved in virulence. To explore genes involved in virulence, we selected all 354 genes and performed PHI-BLAST. We obtained 28 proteins from the PHI-BLAST hits. Thirteen of them play an important role in virulence in other fungi because the corresponding mutants showed reduced virulence, and 15 were annotated as fatal (Supplementary Table S3). These data indicate that these genes may contribute to virulence downstream of VpxlnR in *V. pyri*.

### Identification of Virulence- or Growth-Related Genes Binding to VpxlnR

**Figure 6:**
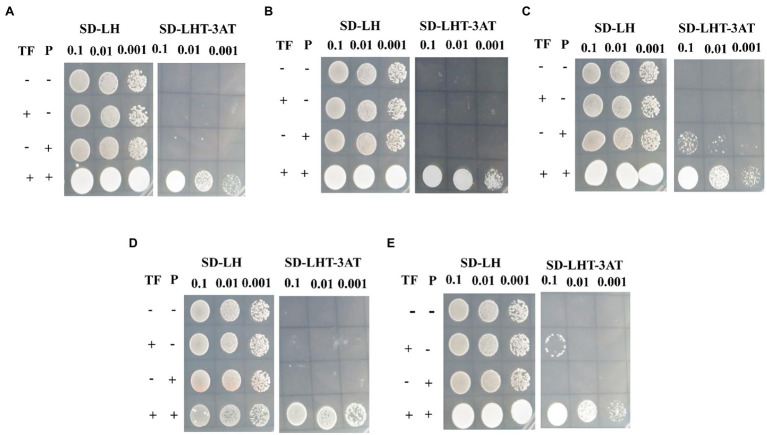
Assessment of *VpxlnR* binding activity using yeast one-hybrid (Y1H). Genes were selected from PHI-BLAST analysis, including **(A)** VP1G_09520: salicylate hydroxylase, **(B)** VP1G_03128: serine/threonine-protein kinase, **(C)** VP1G_06369: alpha-xylosidase, **(D)** VP1G_02856: G-protein beta subunit, and **(E)**. VP1G_03782: acid phosphatase. *VpxlnR* and pHis2::P coexpressed in yeast cells were cultured on SD/−Leu/His plates, and positive clones were screened on SD/−Trp/−Leu/-His plates with 3AT plates. TF: VpxlnR, P: promoter. Empty pGADT7 (−) and empty pHIS2 (−) plasmids, and pGADT7::*VpxlnR***/**empty pHIS2 vector and empty pGADT7/pHIS2::P were used as controls. (+) indicates that the yeast cells expressed *VpxlnR or* contained gene promoters. The liquid cultures (OD_600_ = 0.5) were diluted as shown in the image. Positive yeast clones were formed after culture on SD/−Trp/−Leu/-His/3AT for 3 days, and images were captured.

According to gene annotation and PHI-BLAST analysis, we chose the promoters of the eight genes encoding salicylate hydroxylase (VP1G_09520), alpha-xylosidase (VP1G_06369), G-protein beta subunit (VP1G_02856), acid phosphatase (VP1G_03782), putative phosphotransferases (VP1G_03516), and three serine/threonine-protein kinases (VP1G_03128, VP1G_04075, and VP1G_10966) for further characterization. To confirm whether VpxlnR binds to the promoter regions of virulence- or growth-related genes *in vivo*, we performed Y1H assays. The Y1H results showed that VpxlnR could bind to the promoter regions of the genes encoding salicylate hydroxylase (VP1G_09520), serine/threonine-protein kinase (VP1G_03128), alpha-xylosidase (VP1G_06369), G-protein beta subunit (VP1G_02856), and acid phosphatase (VP1G_03782; Figure 6), suggesting that VpxlnR may determine virulence or growth by controlling the expression of extracellular proteins and signaling pathways. VpxlnR did not bind to the promoters of the other three genes (data not shown). Furthermore, we predicted that the DNA-binding motifs of all three target genes (VP1G_09520, VP1G_03782, and VP1G_02856) contain the degenerate sequence MBSGTCCGY (Supplementary Table S4). Another two genes (VP1G_03128 and VP1G_06369) contain a motif including a GGC triplet; however, they were not the same as the reported motif interacting with xlnR orthologues (Supplementary Table S4). These results suggest that VpxlnR could interact with motifs consisting of MBSGTCCGY and GGC triplets *in vivo*.

### Expression Levels of the Target Genes Controlled by VpxlnR

**Figure 7:**
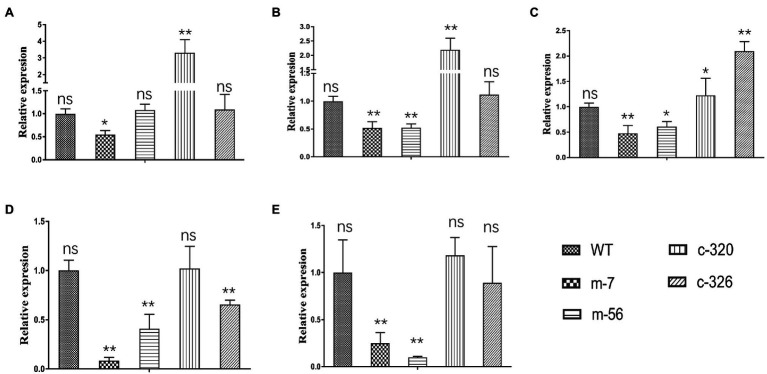
RT–qPCR test of target genes controlled by VpxlnR. **(A)** Salicylate hydroxylase encoding gene (VP1G_09520), **(B)** serine/threonine-protein kinase encoding gene (VP1G_03128), **(C)** alpha-xylosidase encoding gene (VP1G_06369), **(D)** G-protein beta subunit encoding gene (VP1G_02856), and **(E)** Acid phosphatase encoding gene (VP1G_03782). The transcript level of each gene was normalized to actin expression. Each test was repeated three times, and the statistical analysis was performed using one-way ANOVA. ns indicates a value of *p* > 0.05, * indicates a value of *p* < 0.05, and ** indicates a value of *p* < 0.01.

To assess whether the expression levels of five target genes were affected by *VpxlnR* deletion, we analyzed their transcript levels by RT–qPCR. Based on RT–qPCR results, we found that the expression levels of two genes (VP1G_06369 and VP1G_03128) were only reduced in one mutant (m-7) and not in m-56 (Figures 7A,B). These results demonstrated that these two genes (VP1G_06369 and VP1G_03128) might also be controlled by regulators other than xlnR. The other three genes were downregulated in the two mutants (m-7 and m-56; Figures 7C–E), suggesting that VpxlnR could regulate the expression levels of these genes by directly binding gene promoters. These results demonstrated that VpxlnR could positively control the transcript levels of virulence-related genes to contribute to virulence in *V. pyri*.

## Discussion

*Valsa pyri* is a woody pathogen causing trunk canker disease on pear trees in most orchards of northern China. The fungus can infect pear or apple trees, leaves, and fruits, resulting in great yield loss or tree death (Abe et al., 2007; Xu et al., 2018). *Valsa pyri* is a necrotrophic fungus that can penetrate the phloem and xylem. Transcriptomic analysis also showed that cellulose- or pectin-degrading enzyme-encoding genes were significantly upregulated in the infection stage (He et al., 2018). However, cellulase and pectinase were not the key factors determining virulence in *V. mali* (Yin et al., 2015). Previous studies showed that several proteins secreted by *V. mali* function as effectors, which could cause cell death or change the immune system in *Nicotiana benthamiana* (Li et al., 2015b; Feng et al., 2018; Zhang et al., 2019). However, the deletions of these protein-encoding genes in *V. mali* did not lead to a great virulence reduction. Moreover, woody plants are obviously distinguished from herbs, especially trunk diseases. Therefore, studying woody plant pathogens faces greater challenges. VpxlnR and its orthologues generally contain the GAL4 domain and have important roles in controlling xylanolytic activity (van Peij et al., 1998), degradation of the polysaccharides xylan and cellulose (Hasper et al., 2000), and virulence (Calero-Nieto et al., 2007). Due to its potential function in the degradation of xylan and cellulose, which are key chemical components in the trunk or branches of pear trees, VpxlnR may contribute greatly to virulence in *V. pyri*.

The VpxlnR gene contains four introns, and the genome sequence is not well assembled at its location (Yin et al., 2015); thus, the transcriptome *de novo* analysis results provide more clues to identify this gene (Supplementary Figure S1). The xlnR protein predicted in the *V. pyri* genome lost a GAL4 domain. However, our study showed that it contains a GAL4 domain. Additionally, most xlnR orthologues represent one zinc cluster, especially VmxlnR, which shares high identity with VpxlnR. We deduced that xlnR proteins generally contains the GAL4 domain, and those orthologues with the GAL4 domain lost may because of the wrong prediction. Therefore, we think predictions for xlnR orthologues should be rigorous, and the genomes are not sufficient to identify this gene in other fungus.

The main functions of xlnR orthologues in *Aspergillus* spp. are to regulate xylose and other polysaccharides (van Peij et al., 1998; Hasper et al., 2004; Noguchi et al., 2011; Ishikawa et al., 2018; Khosravi et al., 2019). However, AnxlnR was not involved in glucose or sucrose utilization. This result implies that xlnR is involved in many aspects of fungal life. In our study, we found that the growth ratio of the *VpxlnR* deletion mutants was significantly increased on glucose compared with the no-glucose control, but compared with the wild-type, their growth ratio was greatly reduced. Thus, VpxlnR should be a key regulator controlling growth by acquiring glucose from the environment or host. When sucrose was added, the results were similar to those of the control, which indicates that VpxlnR does not participate in sucrose utilization. Similar to a previous study (de Souza et al., 2013; Kowalczyk et al., 2015; Llanos et al., 2019), the colony size of the mutants did not decrease significantly on pectin or cellulose plates compared with Vp297, which implies that the mutants may grow normally in the infection stage because of their abilities to utilize cell wall components such as pectin and cellulose. These outcomes are partially in agreement with previous studies and provide new insights into the function of xlnR orthologues.

Previously, FoxlnR did not affect the virulence of *F. oxysporum* on tomato fruits, but it actually affected the pathogenicity of *F. oxysporum* on tomato roots before 10 dpi (Calero-Nieto et al., 2007). It mainly controls the transcript levels of xylanase-encoding genes (Calero-Nieto et al., 2007). In our study, deficiency of the *VpxlnR* deletion mutants on infection was detected on pear branches or leaves (Figure 3). Moreover, the *VpxlnR* deletion mutants were also sensitive to H_2_O_2_ stress and SA stress in a concentration-dependent manner (Figure 3). Interestingly, the Δ*xlnR* strains of *A. niger* were sensitive to oxidative stress when grown on media supplemented with glucose (Raulo et al., 2016). Additionally, the mutants reduced their abilities to utilize glucose or sucrose (Figure 2B). We believe that host extracellular glucose or sucrose might induce intracellular ROS levels in fungi, which leads to growth limitation of the *xlnR* mutants. In the early infection stage, the extracellular space of the host cell contains glucose, sucrose, and host defense chemicals such as ROS and SA, which may impair infectious growth of the mutants in host tissue. However, when they overcome innate immunity, they can break the host cell wall and use pectin, cellulose, and xylose and then exhibit restored virulence. In brief, the *VpxlnR* deletion mutants exhibit susceptibility to the host defense response, resulting in reduced virulence at the infection stage of *V. pyri*.

Zn2Cys6 TFs control target genes by binding a motif including a CGG triplet (Cho et al., 2013; Luo et al., 2016), while XlnR interacts with promoters containing not only CGG triplets but also TTAGSCTAA motifs (Ishikawa et al., 2018). To broadly explore the target genes of VpxlnR, we obtained all of the promoters containing NRHKGMCCGM in the *V. pyri* genome (Cho et al., 2013; Ishikawa et al., 2018). Eight promoters more than 1,500 bp in length were identified and five of them could interact with VpxlnR in Y1H assays. These results suggest that the motif consisting of MDSGTCCGY is very likely to interact with VpxlnR. Moreover, VpxlnR also recognizes a motif containing GGC triplets, although no specific feature was observed in the 3′ flanking sequence of the GGC triplet. These results extend the binding site of xlnR and provide new clues for studying zinc cluster-type TFs.

Genes directly controlled by VpxlnR contain secreted proteins such as alpha-xylosidase and acid phosphatase, signaling transduction regulators including G-protein beta subunit and serine/threonine-protein kinase, and salicylate hydroxylase (Figure 6). There were also three genes encoding putative serine/threonine-protein kinase KIN28 (VP1G_04075) and GCN2 (VP1G_10966) and putative phosphotransferases (VP1G_03516) that could not interact with VpxlnR, but their transcript levels were significantly reduced in the deletion mutants (Supplementary Figure S4). These results suggest that they might function downstream of VpxlnR in regulating virulence. However, there are very limited reports on the function of these two secreted proteins in fungal virulence. G-protein and its regulators could regulate conidiation, antioxidant capacity, and virulence in *M. robertsii*, *Mucor circinelloides*, *Cryphonectria parasitica*, and *Ustilago maydis* (Segers et al., 2004; Moretti et al., 2017; Tong et al., 2020; Valle-Maldonado et al., 2020). Moreover, the serine/threonine-protein kinase ChSch9 participated in the virulence of *Colletotrichum higginsianum* (Sohail et al., 2021). Therefore, we deduced that VpxlnR responds to H_2_O_2_ stress, virulence, and fruiting body formation by controlling the G-protein and serine/threonine-protein kinase encoding genes. Additionally, whether the two secreted proteins contribute to the virulence of *V. pyri* should be confirmed in the future.

## Data Availability Statement

The original contributions presented in the study are included in the article/Supplementary Material, further inquiries can be directed to the corresponding authors.

## Author Contributions

FH designed the experiments, analyzed the data, and wrote the manuscript. A-MK, FH, and JY finished all most experiments. YZ and FL provided funding for this study. ZF revised the manuscript. The others participated in experiments or manuscript revision. All authors contributed to the article and approved the submitted version.

## Funding

This work was supported by grants from the Anhui Provincial Natural Science Foundation (2008085MC77 and 2008085QC129), the Program of Fujian Key Laboratory for Monitoring and Management of Crop Pests (026052020008), the Major Science and Technology Projects in Anhui Province (No. 2020003a06020009), and the Earmarked Fund for China Agriculture Research System (CARS-28-16).

## Conflict of Interest

The authors declare that the research was conducted in the absence of any commercial or financial relationships that could be construed as a potential conflict of interest.

## Publisher’s Note

All claims expressed in this article are solely those of the authors and do not necessarily represent those of their affiliated organizations, or those of the publisher, the editors and the reviewers. Any product that may be evaluated in this article, or claim that may be made by its manufacturer, is not guaranteed or endorsed by the publisher.

## Footnotes

^1^ https://www.ncbi.nlm.nih.gov/orffinder/

^2^ http://pfam.xfam.org/

## Supplementary Material

Supplementary Figure S1 Distinct sites of the sequences predicted in transcriptomics and genomics of *V. pyri*. VP1G_03432 was characterized using *V. pyri* genomics. The sequence of VpxlnR was obtained from *de novo* transcriptomics. The line shown in the figure indicates the gaps of the two sequences.

Supplementary Figure S2 Constructs for VpxlnR replacement and complementation. VpxlnR deletion constructs were generated by triple joint PCR amplification (described in the “Materials and Methods” section). The arrows indicate primer sites. The numbers (1–12) represent the primers (Supplementary Table S5) used for deletion construct generation and genomic PCR identification.

Supplementary Figure S3 The mutant was confirmed by genomic PCR and RT–qPCR. **(A)** Genomic PCR identification for fragments in replacement sites. Primer pairs with numbers are shown in the right panel, and numbers are the same as in Supplementary Figure S1. WT, wild-type strain (Vp297); m-7 and m-56, VpxlnR deletion mutants; C-320 and C-236, VpxlnR complementation strains. **(B)** VpxlnR expression level in deletion mutant. Transcript levels were quantified by RT–qPCR and normalized to *actin* gene expression. Each test was repeat three times. The relative transcript level of the gene at time point 0 was set as 1.0. The data were analyzed using one-way ANOVA. ns indicates a value of *p* > 0.05, ** indicates a value of *p* < 0.01.

Supplementary Figure S4 RT–qPCR test of nontarget genes controlled by VpxlnR. **(A)** Serine/threonine-protein kinase KIN28-encoding gene (VP1G_04075); **(B)** serine/threonine-protein kinase GCN2-encoding gene (VP1G_10966); **(C)** putative phosphotransferase-encoding gene (VP1G_03516). The transcript level of each gene was normalized to actin expression. Each test was repeated three times, and the data were analysed using one-way ANOVA. ns indicates a value of *p* > 0.05, * indicates a value of *p* < 0.05, and ** indicates value of *p* < 0.01.
