# Supplementary figures and images for: The Transcription Factor VpxlnR Is Required for the Growth, Development, and Virulence of the Fungal Pathogen Valsa pyri
Source: Front Microbiol. 2022 Mar 3;13:784686. doi: 10.3389/fmicb.2022.784686 (PMC8928461; doi:10.3389/fmicb.2022.784686)

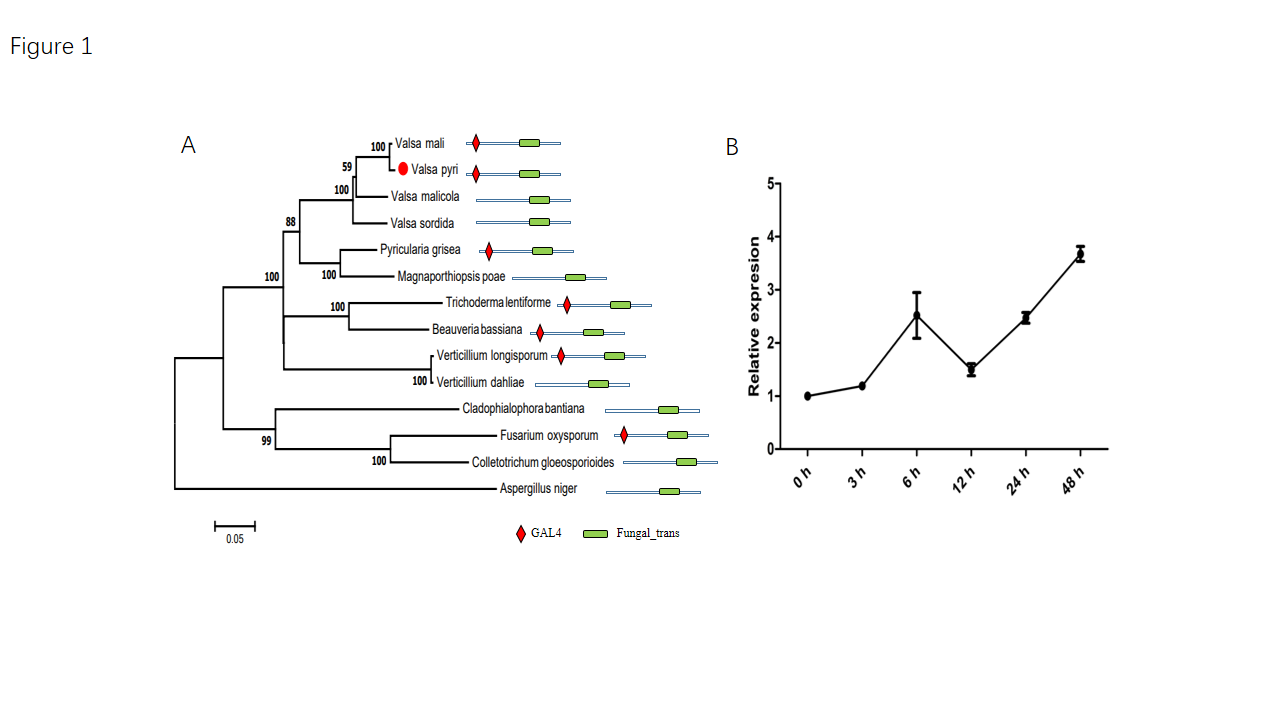

Supplement: Supplementary Figure S1 — Distinct sites of the sequences predicted in transcriptomics and genomics of V. pyri. VP1G_03432 was characterized using V. pyri genomics. The sequence of VpxlnR was obtained from de novo transcriptomics. The line shown in the figure indicates the gaps of the two sequences. [file Image_1.TIF]

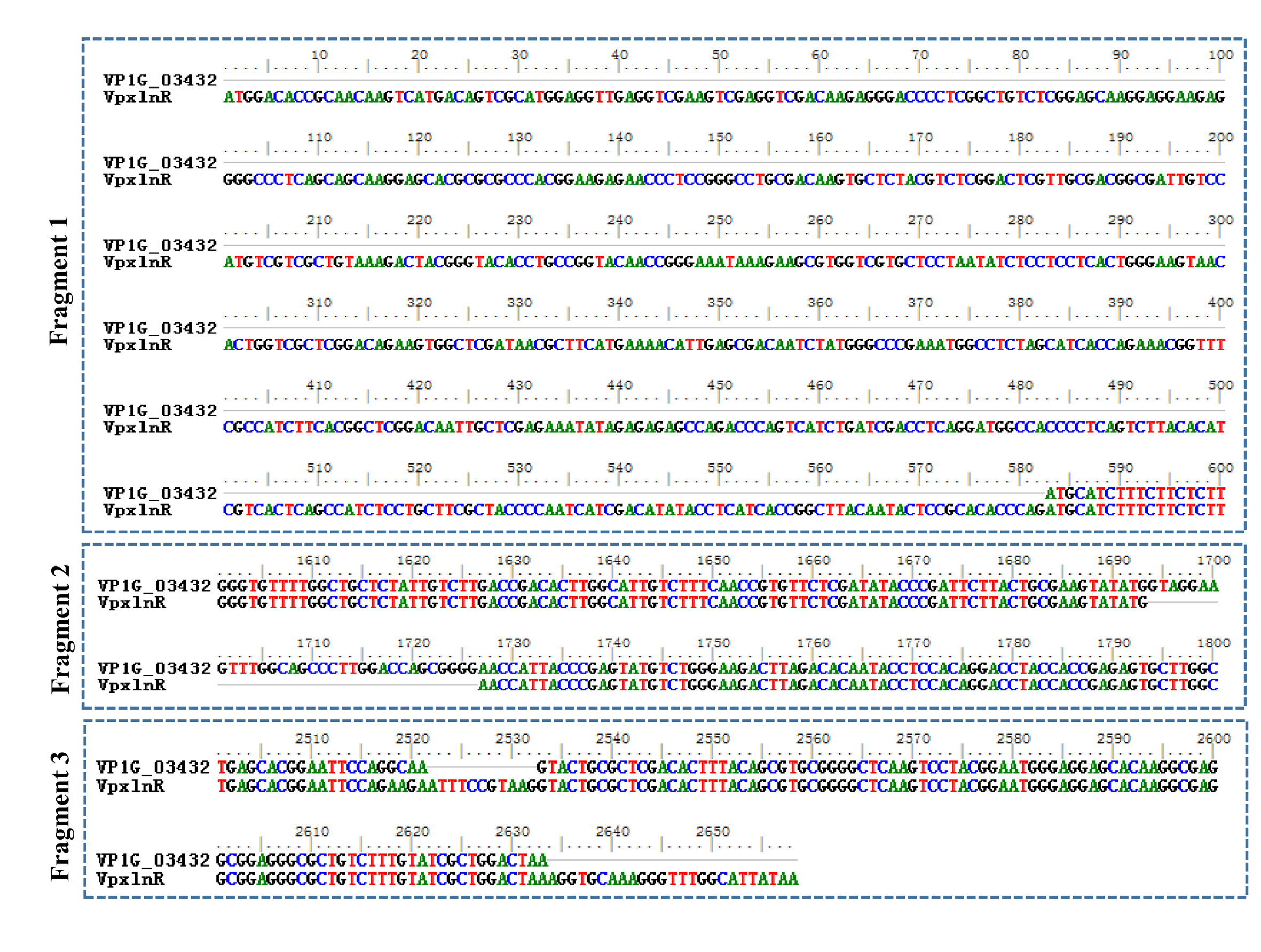

Supplement: Supplementary Figure S2 — Constructs for VpxlnR replacement and complementation. VpxlnR deletion constructs were generated by triple joint PCR amplification (described in the “Materials and Methods” section). The arrows indicate primer sites. The numbers (1–12) represent the primers (Supplementary Table S5) used for deletion construct generation and genomic PCR identification. [file Image_2.TIF]

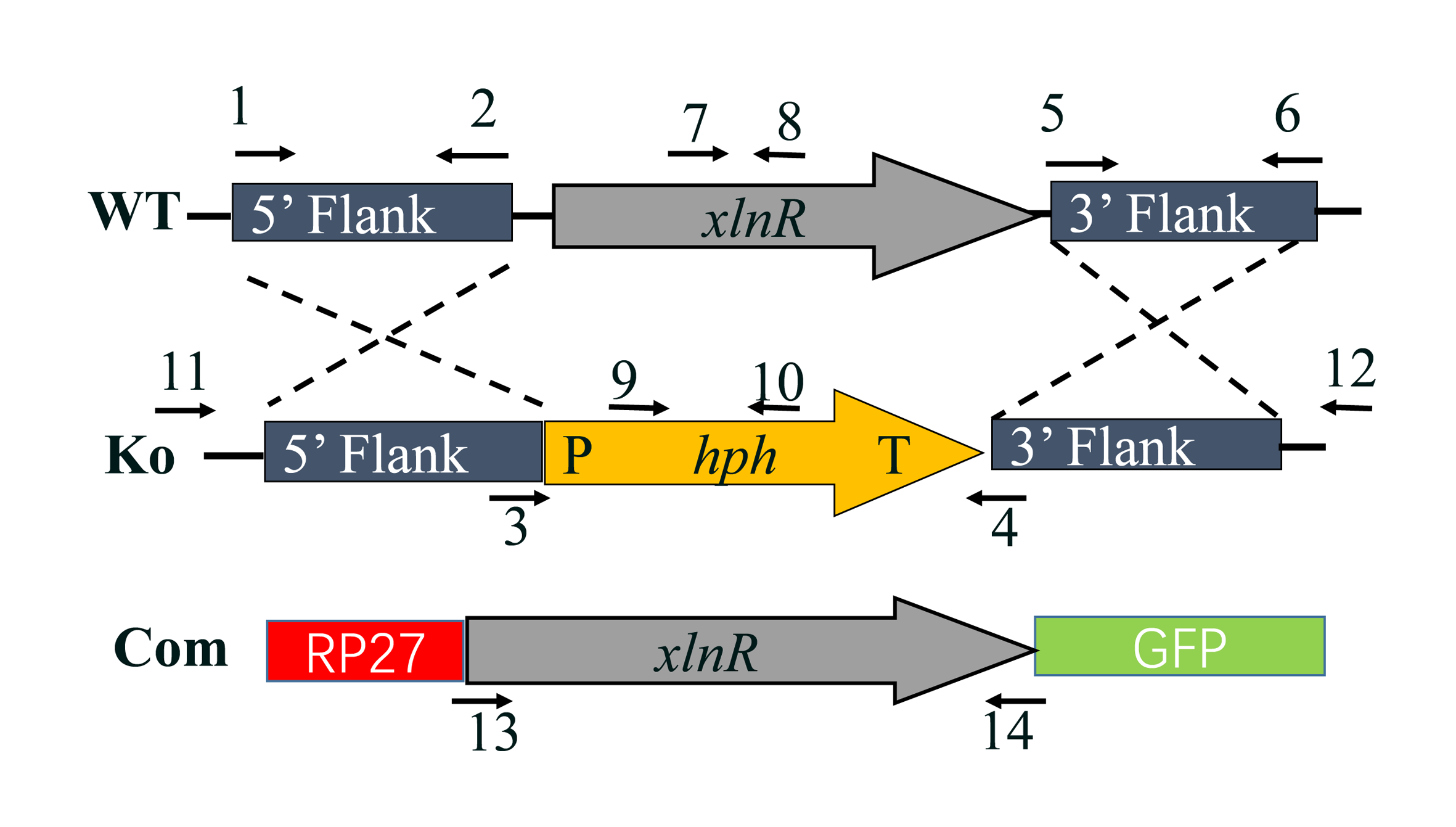

Supplement: Supplementary Figure S3 — The mutant was confirmed by genomic PCR and RT–qPCR. (A) Genomic PCR identification for fragments in replacement sites. Primer pairs with numbers are shown in the right panel, and numbers are the same as in Supplementary Figure S1. WT, wild-type strain (Vp297); m-7 and m-56, VpxlnR deletion mutants; C-320 and C-236, VpxlnR complementation strains. (B) VpxlnR expression level in deletion mutant. Transcript levels were quantified by RT–qPCR and normalized to actin gene expression. Each test was repeat three times. The relative transcript level of the gene at time point 0 was set as 1.0. The data were analyzed using one-way ANOVA. ns indicates a value of p > 0.05, ** indicates a value of p < 0.01. [file Image_3.TIF]

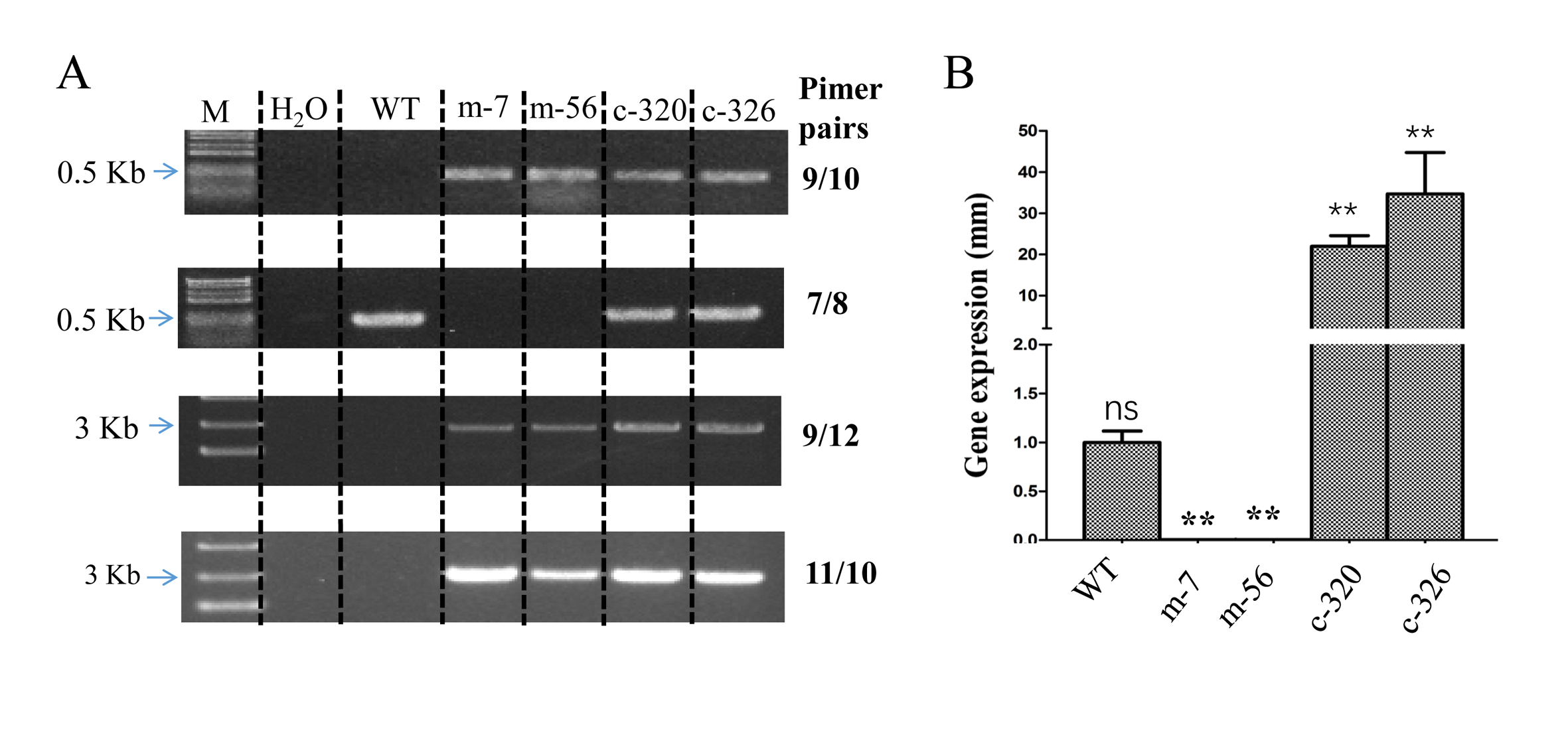

Supplement: Supplementary Figure S4 — RT–qPCR test of nontarget genes controlled by VpxlnR. (A) Serine/threonine-protein kinase KIN28-encoding gene (VP1G_04075); (B) serine/threonine-protein kinase GCN2-encoding gene (VP1G_10966); (C) putative phosphotransferase-encoding gene (VP1G_03516). The transcript level of each gene was normalized to actin expression. Each test was repeated three times, and the data were analysed using one-way ANOVA. ns indicates a value of p > 0.05, * indicates a value of p < 0.05, and ** indicates value of p < 0.01. [file Image_4.TIF]

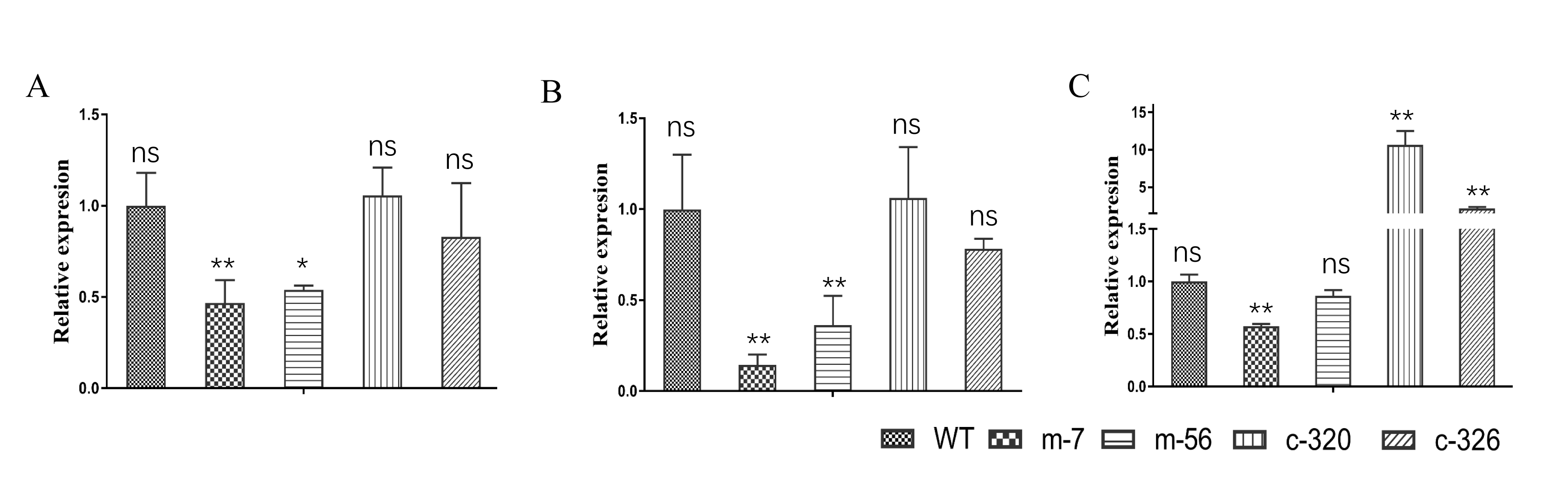

Supplement: Supplementary file 5 [file Image_5.TIF]
