# Supplementary material for: The Transcription Factor VpxlnR Is Required for the Growth, Development, and Virulence of the Fungal Pathogen Valsa pyri
Source: Front Microbiol. 2022 Mar 3;13:784686. doi: 10.3389/fmicb.2022.784686 (PMC8928461; doi:10.3389/fmicb.2022.784686)
Supplement: Supplementary file 10 [file Table_5.DOCX]

**TABLE S5. The oligonucleotides used in this study**

| No. | Product | utilization | **Sequence (5’ to 3’)** |
| --- | --- | --- | --- |
| 1 | 5’-flanking F | 5’ flanking | TCGTCGGATTGCGAACAT |
| 2 | 5’-flanking R |  | ACCAGCCAGCCAACAGCTCCCTGGACAACCCAAAATAGGC |
| 3 | 3’-flanking F | 3’flanking | ATACGCAAACCGCCTCTCCC CAGTGGTTGGGTAGTTCTCC |
| 4 | 3’-flanking R |  | TATTATCGCCACGTCGTCTA |
| 5 | Hph box-F | Hph box | GCCTATTTTGGGTTGTCCAGGGAGCTGTTGGCTGGCTGGT |
| 6 | Hph box-R |  | GGAGAACTACCCAACCACTGGGGAGAGGCGGTTTGCGTAT |
| 7 | deletion region | Partial *vpxlnR gene* | ATACCTCCACAGGACCTACCA |
| 8 | deletion region |  | GCCCTCACAATCGTCTCAC |
| 9 | Hph fragment-F | Part of Hph gene | ATCTCATCTCAAACCACGGA |
| 10 | Hph fragment-R |  | ATGTAGGCACGGACAGCAC |
| 11 | Outer F | outer 5’ flanking primer | TCGCCGCAGTGTATGGAA |
| 12 | Outer R | outer 3’ flanking primer | CGTGCAAGAAGTCGGGTC |
| 13 | VpXlnR ORF F | For complementary strain | CACCATCACCATCACTCGAG  ATGGACACCGCAACAAGT |
| 14 | R |  | TCGCCCTTGCTCACCCTCGAGTTTTATAATGCCAAACCCTTTG |
| 15 | VpXlnR F | For qPCR test | CCTGCGACAAGTGCTCTACG |
| 16 | R |  | GCACGACCACGCTTCTTTATT |
| 17 | VpxlnR ORF F | PGADHt7 | gtaccagattacgctcatatgATGGACACCGCAACAAGT |
| 18 | R |  | actggcctccatggccatatgTTATAATGCCAAACCCTTTG |
| 19 | VP1G_03128 F | pHis2 | ctatagggcgaattcccgggGGAGGACTGATGGTAAGCG |
| 20 | R |  | gaacgcgtgagctccccgggAAGAGGTAGCAGGGCGATA |
| 21 | VP1G_03516 F | pHis2 | ctatagggcgaattcccgggGGTATGAGTCCTCGCCGTAT |
| 22 | R |  | gaacgcgtgagctccccgggAGGGTTCACCCAGATTTAGC |
| 23 | VP1G_06369 F | pHis2 | ctatagggcgaattcccgggATGACCGACGATGGACTTGA |
| 24 | R |  | gaacgcgtgagctccccgggGTTCCTGCCTGTTAGCCTTG |
| 25 | VP1G_02856 F | pHis2 | ctatagggcgaattcccgggTCACAGCATGGTCGTAAATG |
| 26 | R |  | ctatagggcgaattcccgggTCACAGCATGGTCGTAAATG |
| 27 | VP1G_04075 F | pHis2 | ctatagggcgaattcccgggTTCGCATTCGTGTCCCTCA |
| 28 | R |  | gaacgcgtgagctccccgggCGCCAACCCACAAATCAAC |
| 29 | VP1G_10966 F | pHis2 | ctatagggcgaattcccgggCCCCATCACCACCGTGTA |
| 30 | R |  | gaacgcgtgagctccccgggGTCGCCAAACTTCCGTCT |
| 31 | VP1G_09520 F | pHis2 | ctatagggcgaattcccgggAGTCGCCTGGTAGTCGTGG |
| 32 | R |  | gaacgcgtgagctccccgggCAAGGGTACTGGAGGGTTGTA |
| 33 | VP1G_03782 F | pHis2 | ctatagggcgaattcccgggCACTTCCAGTGCCAAGGAT |
| 34 | R |  | gaacgcgtgagctccccgggAGGACGAGCTACATGAGGG |
| 35 | VP1G_03128 F | qPCR | TGGACATCTGGGCATTTGG |
| 36 | R |  | TCTCGTGGGTCTTCGGTCA |
| 37 | VP1G_03516 F | qPCR | TGACCGACGAGATGGAAAGG |
| 38 | R |  | CCAGGTCAGCGTCAAGCAGT |
| 39 | VP1G_02856 F | qPCR | TACATCAACACCGTCACCATCTC |
| 40 | R |  | TGGCGTTCAGGGAGTACAGG |
| 41 | VP1G_03782 F | qPCR | ACGGCGTCTATTGTGGATAACTC |
| 42 | R |  | CCTCATCAAGGATACTGTGGCTC |
| 43 | VP1G_10966 F | qPCR | TACCTACACCCAGCATCTACCCT |
| 44 | R |  | GAATTCGTCGATTTAGCCAACA |
| 45 | VP1G_04075 F | qPCR | CCCAACATCATCTCCCTCCTC |
| 46 | R |  | TACCGTATGCCCTTGTCGTCT |
| 47 | VP1G_09520 F | qPCR | GCGGATGAGATGGGTTATGG |
| 48 | R |  | ATCCTCGTCTTGCCCCTGTA |
| 49 | VP1G_06369 F | qPCR | AGGTGGACTGCCTTGAAGAATC |
| 50 | R |  | CGTTGGGTCGTCAGGAAACT |
